# Reach, uptake, and psychological outcomes of two publicly funded internet-based cognitive behavioural therapy programs in Ontario, Canada: an observational study

**DOI:** 10.1186/s13033-024-00651-9

**Published:** 2024-11-07

**Authors:** Bilal Noreen Khan, Rebecca H. Liu, Cherry Chu, Blanca Bolea-Alamañac, Megan Nguyen, Serena Thapar, Roz Fanaieyan, Marisa Leon-Carlyle, Mina Tadrous, Paul Kurdyak, Anne O’Riordan, Maggie Keresteci, Onil Bhattacharyya

**Affiliations:** 1 Women’s College Hospital, 76 Grenville St, Toronto, ON M5S 1B2 Canada; 2 Institute of Health Policy, Management and Evaluation, University of Toronto, Toronto, ON Canada; 3 Department of Psychiatry, University of Toronto, Toronto, ON Canada; 4 Department of Psychology, McGill University, Montreal, QC Canada; 5 Leslie Dan Faculty of Pharmacy, University of Toronto, Toronto, ON Canada; 6 Temerty Faculty of Medicine, University of Toronto, Toronto, ON Canada; 7 Centre for Addiction and Mental Health, Toronto, ON Canada; 8 Patient Advisors Network, Toronto, ON Canada; 9 Department of Family and Community Medicine, University of Toronto, Toronto, ON Canada

## Abstract

**Background:**

Access to traditional mental health services in Canada remains limited, prompting exploration into digital alternatives. The Government of Ontario initiated access to two internet-based cognitive behavioral therapy (iCBT) programs, LifeWorks AbilitiCBT and MindBeacon TAiCBT, for adults with mental health issues.

**Methods:**

An uncontrolled observational study utilizing secondary retrospective program data was conducted to evaluate the reach, uptake, and psychological symptom changes among participants engaging with either iCBT program.

**Results:**

Between May 2020 and September 2021, 56,769 individuals enrolled in LifeWorks AbilitiCBT, and 73,356 in MindBeacon TAiCBT. However, substantial exclusions were made: 56% of LifeWorks participants and 68% of MindBeacon participants were ineligible or failed to initiate treatment. Consequently, 25,154 LifeWorks participants and 23,795 MindBeacon participants were included in the analysis. Of these, 22% of LifeWorks and 26% of MindBeacon participants completed over 75% of iCBT treatment. On average, LifeWorks participants received 13 ± SD 7.1 therapist messages and sent 5 ± SD 10.3 messages, while MindBeacon participants received 25 ± SD 20.7 therapist messages and sent 13 ± SD 16.4 messages. LifeWorks included synchronous therapist contact averaging 1.4 ± SD 1.9 h per participant, while MindBeacon was purely asynchronous. Baseline severity of anxiety (37%) and depression symptoms (22%) was higher for LifeWorks participants compared to MindBeacon participants (24% and 10%, respectively). Clinically significant changes in anxiety and depression scores were observed: 22% of LifeWorks and 31% of MindBeacon participants exhibited reliable recovery in PHQ-9 scores, while 26% of LifeWorks and 25% of MindBeacon participants demonstrated reliable recovery in GAD-7 scores.

**Conclusion:**

In conclusion, iCBT programs show promise for engaged participants with varying levels of severity in anxiety and depression symptoms. Future iterations of iCBT should consider adopting a broad entry criterion to iCBT programming to increase accessibility, especially for those with severe symptoms, alongside integrated intake care pathways, and potential payment structure adjustments for iCBT service providers. Taken all together, these factors could temper high dropout rates post-intake assessment. This evaluation underscores the potential and value of digital mental health interventions for individuals with mild to severe anxiety or depression symptoms, emphasizing the importance of addressing participant dropout.

## Introduction

Over 5.3 million Canadians require some type of mental health care every year, out of which almost half reported their mental health needs were not met [1, 2]. While effective treatment options for mental health disorders exists, access to mental health services is limited due to long wait and referral times, geographical inequities, shortage of mental health professionals, cost of mental health services, lack of integration with other services, cultural and language barriers, and stigma related to help seeking behaviours [1, 3]. Given the increasing prevalence of mental health disorders, population-level prevention, promotion, and early intervention are required to minimize lasting negative impacts [4–6]. At the onset of the COVID-19 pandemic, a rise in virtual care was apparent which provided a unique opportunity to leverage existing digital platforms to address the increase in mental health concerns and reduce strain on an already over-burdened mental health care system [7, 8]. Digital mental health interventions like internet-based cognitive behavioral therapy (iCBT) were deployed at a large-scale, offering an accessible, preventive, and first-line treatment option [8]. As a general psychosocial intervention, iCBT can be an effective treatment for mental health conditions including mood disorders, social anxiety, panic disorders, phobias, substance use disorders, and obsessive-compulsive disorder [9–11]. In May 2020, the Ontario government provided funding to scale up and expand virtual mental health service offerings to the general public through two therapist-assisted iCBT programs: LifeWorks AbilitiCBT and MindBeacon TAiCBT [12].

It was imperative to understand who accessed and had change in symptoms to inform future policy and investment decisions. Several clinical trials suggest that iCBT is effective and broadly accessible [10, 11, 13], with growing evidence of successful iCBT implementation as part of routine care in Canadian provinces and other jurisdictions [14–18]. Small randomized controlled trials of iCBT have higher levels of adherence and lower dropout rates often provided to targeted and more symptomatic sub-populations compared to programs that are offered directly to the general population [19, 20]. RCTs can assess the impact of interventions in a more controlled setting, although they are not well suited to evaluate real-world implementation [21].

Real world implementations of iCBT exist such as Improved Access of Psychological Therapies (IAPT) in the United Kingdom, MindSpot, NewAccess, and This Way Up in Australia, and The Online Therapy Unit in Alberta, Canada [14–18]. Although these real-world implementations of iCBT differ in how they were offered whether as standalone courses tailored to address specific mental health issues or for sub-population groups (This Way up, MindSpot, Online Therapy Unit) or as part of stepped care models with a variety of low to high intensity mental health services (NewAccess and IAPT). It is noteworthy to mention that LifeWorks AbilitiCBT and MindBeacon TAiCBT were standalone guided programs but not part of a stepped care pathway. Evidence to inform policy decisions require innovative approaches using readily available data for timely, cost-effective, and robust evaluations [21]. This third-party evaluation intends to add to the growing literature with a head-to-head comparison of two standalone iCBT programs in a large and differentiated population in Ontario, Canada with the following objectives: [1] To determine reach of these iCBT programs to Ontario’s population [2], To describe uptake, completion, and engagement in both iCBT programs, and [3] To explore changes in psychological symptoms of individuals that initiated treatment.

## Methods

### Description of programs

LifeWorks AbilitiCBT and MindBeacon TAiCBT were predominantly designed as self-referral programs, advertised through Ontario’s provincial website, and through the LifeWorks and MindBeacon websites respectively. Referral through several participating hospitals across Ontario was another referral pathway to the programs though less common. LifeWorks AbilitiCBT and MindBeacon TAiCBT were available to adults in English and French, and are based on Beck’s Cognitive Triad, employing cognitive behavioural therapy principles and techniques [22]. Key program features included an intake assessment to assess primary mental health issues, patient-guided and goal-oriented program components, and communication with a trained therapist during treatment. Some mental health concerns, like pandemic-related anxiety and trauma support, are not classified in the Diagnostic and Statistical Manual of Mental Disorders, 5th edition (DSM-IV) [23]. These concerns were only assessed by LifeWorks AbilitiCBT. Prior to commencing either iCBT program, intake assessments obtained baseline PHQ-9 and GAD-7 scores to determine suitability and identify individuals at imminent risk to themselves or others, or those requiring higher levels of care. Such individuals were promptly referred to immediate medical care or existing mental health and substance use services. Ongoing assessments of PHQ-9 and GAD-7 scores and client communication with therapists, enabled continuous evaluation for suicidality and the need for higher levels of care. Key differences between the two programs were how and by whom intake assessment was administered, type of therapist contact provided to participants (asynchronous vs. synchronous communication) and programmatic components based on identified mental health issues. Components of the two programs can be found in Table 1. More information on the clinical features and specific program components can be found on LifeWorks (https://go.lifeworks.com/en-ca/icbt-on) and MindBeacon websites ((https://www.mindbeacon.com/guided-cbt-programs).

**Table 1:** Program characteristics of LifeWorks AbilitiCBT and MindBeacon TAiCBT

Program Characteristics	Lifeworks AbilitiCBT	MindBeacon TAiCBT
**Eligibility Criteria**	• Ontario resident • Age 18 years or older • Complete information provided in intake assessment	
**Languages offered**	• English • French	
**Program Components**	**10–12 Modules** • 10 modules, with 2 extra modules for those with symptoms of post-traumatic stress disorder (PTSD) • Tailored readings and activities	**7 to 16 Playlists** • Additional playlists as required • Tailored readings and activities • Modular based on condition and participant-needs and preferences
**Program duration**	Estimated 10 weeks or 70 days to complete	Estimated 12 weeks or 84 days to complete
**iCBT Therapists** **Note: Same clinician throughout program**	Registered mental health professionals • Social workers • Psychotherapists • Psychologist Including a Master’s level education and at least two years of post-Master’s clinical experience	Registered mental health professionals • Social workers • Psychotherapists • Psychologist
**Type of therapist assistance (e.g.**,** check-in**,** follow-ups)**	• **Asynchronous** in-application messaging • Synchronous* scheduled in-application video and/or audio calls *Mandatory synchronous in-application video or audio call with therapist for intake assessment upon entry to program,* thereafter follow-up modality mutually decided upon and scheduled by participant and therapist*	• **Asynchronous** in-application messaging)

### Design and ethics

A third-party evaluation was conducted comparing two iCBT programs using secondary retrospective data from individuals who registered for iCBT between May 2020 till September 2021. This study only included individuals who consented to having their data used for evaluation or research related to the programs. A restricted dataset was provided by LifeWorks, thus the number of individuals who initiated LifeWorks AbilitiCBT without consent is unknown. However, 3,460 individuals did not consent for their data to be used for evaluation and initiated MindBeacon TAiCBT. A retrospective study with two treatment groups was conducted. The Women’s College Hospital Institute for Health Care Solution and Virtual Care (WIHV) signed data sharing agreements with LifeWorks AbilitiCBT and MindBeacon TAiCBT respectively to obtain their data for evaluation and research purposes. The Centre for Digital Health Evaluation (CDHE) received approval from the Assessment Process for Quality Improvement Projects (APQIP) at Women’s College Hospital and obtained an exemption letter from the Women’s College Hospital Research Ethics Board under REB # 2021-0057-E in compliance with the Tri-Council Policy Statement (TCPS2).

### Engagement

Both program platforms captured data on participant engagement, including patient-therapist communication, program completion rate, and duration of program. Patient-therapist communication was measured with two variables: total number of in application messages sent by each participant to their therapist and total number of in application messages received from therapist. In addition to these two variables, LifeWorks AbilitiCBT collected total number of clinical hours spent by therapist communicating with each patient. Program completion rate was calculated dividing the number of programmatic components a participant completed by the number of programmatic components assigned to a participant. Program completion was calculated and recoded into a categorical variable. It is important to note that MindBeacon TAiCBT had a unique number and combination of programmatic components provided to each participant dependent on their primary mental health issue identified during the intake assessment along with their changing needs and preferences throughout treatment. While completion rates were calculated for both iCBT programs, it is important to acknowledge the different programmatic features as outlined in Table 1. Consequently, completion rates may reflect variations in how participants engage with the distinct features of each program. While these completion rates offer insights, direct comparisons should be approached with consideration of the nuanced programmatic differences. Lastly, duration in program was calculated for each participant that initiated iCBT by subtracting the date and time when they initiated the program by the date and time when they exited or last interacted with the program.

### Primary outcome measures

Primary outcome measures for the iCBT programs were change in the 9-Item Patient Health Questionnaire (PHQ-9) [24] and the Generalized Anxiety Disorder 7-Item Scale (GAD-7) [25]. Each participant completed the PHQ-9 and GAD-7 scales pre- and post-treatment. The pre-measurement was self-reported during the intake assessment, while the post-measurement comprised the final scores provided by participants before leaving the program. The PHQ-9 is a self-administered validated questionnaire comprising of nine statements that are based directly on the nine diagnostic criteria for major depressive disorder in the Diagnostic and Statistical Manual of Mental Disorder, 4th edition (DSM-IV) [23]. Research has shown the PHQ-9 to have good internal consistency, sensitivity to change, and strong correlation with subsequent major depression diagnosis [26]. Scores can range from 0 to 27, with a score between 0 and 4 indicating minimal depression, a score between 5 and 9 indicating mild depression, a score between 10 and 14 indicating moderate depression, a score of 15–19 indicating moderately severe depression, and a score between 20 and 27 indicating severe depression [24]. While the PHQ-9 tool is intended to assist health care professionals in screening for and identify depressive symptoms, it is not a substitute for a comprehensive diagnosis by a trained clinician [27]. Scores of 10 and above on the PHQ-9 were categorized as clinical cases [28]. The GAD-7 is a self-administered validated questionnaire comprising of 7 statements that are sensitive to the presence of generalized anxiety disorder along with social phobia, panic disorder and post-traumatic stress disorder [25]. Scores can range from 0 to 21, with a score between 1 and 4 indicating minimal anxiety, a score between 5 and 9 indicating mild anxiety, a score between 10 and 14 indicating moderate anxiety, and a score of 15–21 indicating severe anxiety [25]. Research has shown the GAD-7 to have good reliability and internal consistency, as well as convergent validity [29]. The GAD-7, like the PHQ-9, provides only probable diagnoses that should be confirmed with further evaluation by a trained clinician [25]. Scores of 8 and above on the GAD-7 were categorized as clinical cases [28].

### Statistical analysis

Only participants meeting the following criteria were included in the full analysis: completion of an intake assessment, eligibility for iCBT as a suitable treatment, consent for research use of self-reported data, and initiation of iCBT through playlist or module completion. Participants failing to meet any of these criteria were excluded from the full analysis. In the dataset, 25,145 LifeWorks and 23,795 MindBeacon participants met the inclusion criteria, while 31,624 LifeWorks and 49,561 MindBeacon participants were excluded. No missing values were imputed prior to analysis. Descriptive statistics were conducted on demographic and clinical features of participants as well as for the three measures of engagement, including patient-therapist communication, program completion, and duration in program. Next, generalized estimating equation (GEE) models were fitted with PHQ-9 and GAD-7 scores as the dependent variables, and time (1 = pre and 2 = post), baseline PHQ-9 baseline GAD-7, and program completion (1 = Less than 25%, 2 = 26–50%, 3 = 51-74%, and 4 = More than 75%) as the independent variables. An unstructured correlation matrix and maximum likelihood estimation were used, and a Gaussian distribution with a linear response was specified as the primary outcome variables were symmetrical. Clinically significant changes were examined through percentage symptom changes from baseline, with *Hedges g* effect sizes calculated to measure magnitude of difference between pre- and post- scores using GEE estimated marginal means. Previously determined clinically reliable change cut-offs based on the Improving Access to Psychological Therapies (IAPT) program (GAD-7 change ≥ 4 and PHQ-9 change ≥ 6) were used to categorize each participant’s change in primary outcomes scores [28]. Reliable deterioration rate was calculated based on participants who showed reliable increase between pre and post scores (PHQ-9 score more than 6 and GAD-7 score more than 4) [28]. Recovery rate was calculated based on participants who were considered clinical cases at baseline but were not clinical cases at the end of the program [28]. Clinical cases were defined as participants exceeding accepted clinical thresholds for GAD-7 (≥ 8) or PHQ-9 (≥ 10) [28]. Data was analyzed using Stata BE version 17.

## Results

### Participant characteristics

**Fig. 1:**
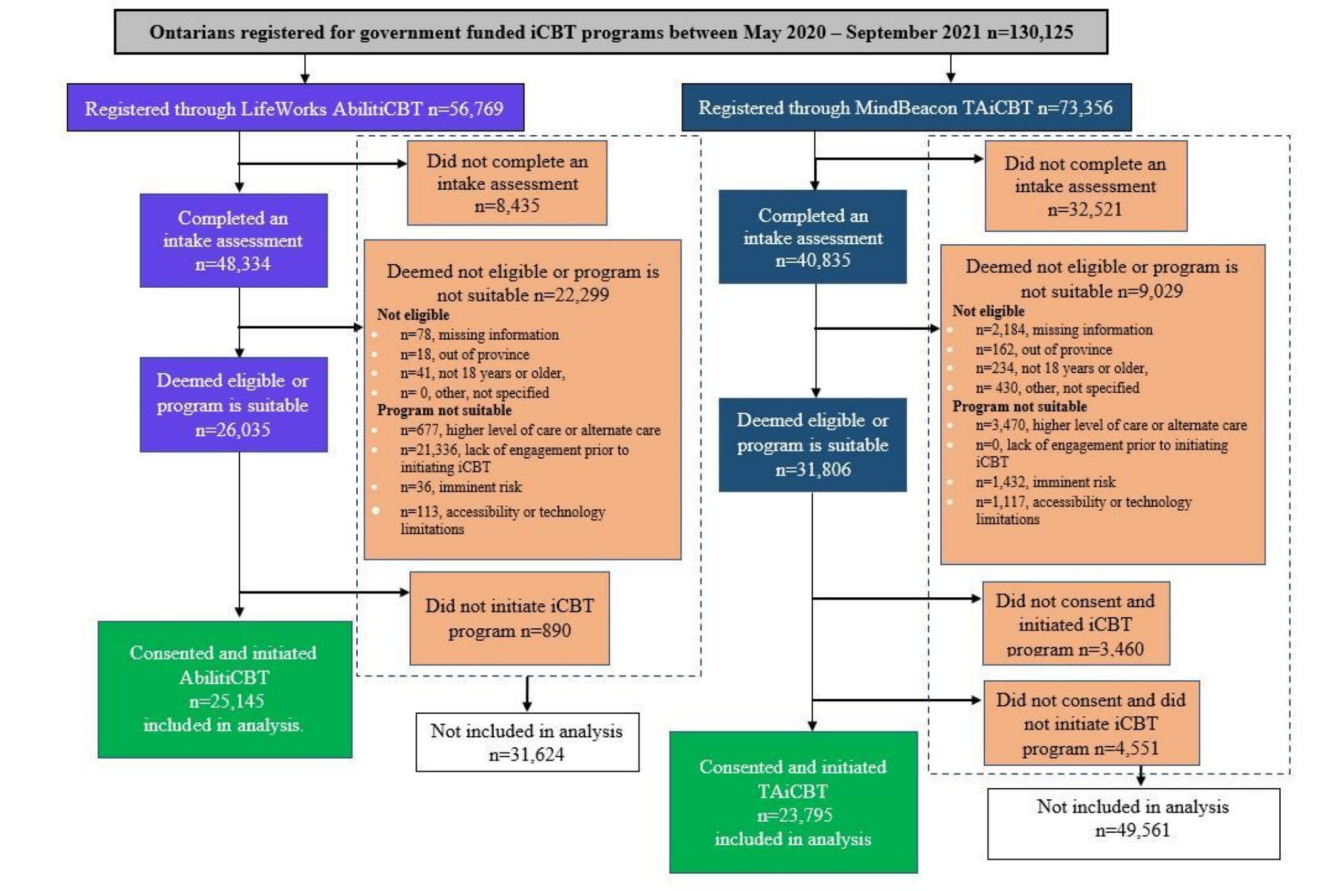
Participant Flow Diagram & Cohort Inclusion. Illustrates the progression of LifeWorks and MindBeacon participants from registration through eligibility assessment, iCBT suitability determination, and research data consent, concluding with the formation of the iCBT-initiated participant cohort

Figure 1 details participant flow and final cohorts of participants included in full analysis. Between May 2020 and September 2021, 56,769 people registered for LifeWorks AbilitiCBT and 73,356 people registered for MindBeacon TAiCBT. Of those who registered, only individuals who completed the intake assessment, met eligibility and suitability, consented for their data to be use, and initiated iCBT by completing one or more playlist or module remained in the cohort, leading to a total of 25,145 (44.3%) participants in LifeWorks AbilitiCBT and 23,795 (32.4%) participants in MindBeacon TAiCBT that initiated iCBT and were included in our analyses. Among participants analyzed, 9% (*n* = 2,154) of LifeWorks and 10% (*n* = 2,360) of MindBeacon participants were missing pre-post self-reported PHQ-9 scores. Similarly, 14% (*n* = 3,409) of LifeWorks and 10% (*n* = 2,350) of MindBeacon participants were missing pre-post self-reported GAD-7 scores. Statistical techniques to impute for missing data were not employed.

Demographic characteristics of participants who initiated iCBT through LifeWorks AbilitiCBT and MindBeacon TAiCBT are shown in Table 2 along with provincial or national estimates provided for comparison where available. For both programs, participants were on average between 35.5 ± SD 12.3 years old, predominantly made up of women (LifeWorks [74.7%], MindBeacon [76.3%]), who identified as ‘White’ (LifeWorks [40.5%], MindBeacon [70.8%]), and who resided in large and urban population centres (LifeWorks [65.4%], MindBeacon [71.1%]). Participants were mostly self-referred (LifeWorks [98.7%], MindBeacon [99.6%]), not healthcare workers (LifeWorks [86.8%], MindBeacon [83.8%]), and not post-secondary students (LifeWorks [66.1%], MindBeacon [52.0%]). The distribution of the participant pool was similar to national/provincial estimates with regards to age and location of residence, although it had a higher proportion of those whom identified as woman, ‘White’, healthcare workers, and post-secondary students compared to national/provincial estimates. Individuals characterized as having generalized anxiety disorder represented the largest group for both LifeWorks (57.9%) and MindBeacon (38.5%).

**Table 2:** Demographics, baseline measures, and engagement of participants that initiated iCBT between May 2020 and September 2021

	LifeWorks AbilitiCBT: All participants who initiated iCBT (*n* = 25,145)	MindBeacon TAiCBT: All participants who consented and initiated iCBT (*n* = 23,795)	National or provincial estimates (if available)
**Age**			
Mean (SD)	36 (12.3)	35 (12.3)	41.0 [40]
Range	18–84	18–92	-
**Gender**			
Woman	74.7% (*n* = 18,788)	76.3% (*n* = 18,155)	50.7% [41]
Man	22.4% (*n* = 5,638)	22.4% (*n* = 5,323)	49.3% [41]
Sexual and gender minorities*	1.8% (*n* = 440)	0.9% (*n* = 210)	-
Prefer not to answer	1.1% (*n* = 279)	0.4% (*n* = 107)	-
Did not report	-	-	-
**Race/ethnicity**			
White	40.5% (*n* = 10,190)	70.8% (*n* = 16,850)	61.6% [42]
South Asian	6.7% (*n* = 1,673)	7.7% (*n* = 1,830)	8.9% [42]
Prefer not to answer	4.3% (*n* = 1,068)	-	-
Other	3.5% (*n* = 884)	3.4% (*n* = 806)	-
Black	3.2% (*n* = 795)	3.6% (*n* = 859)	3.1% [42]
Chinese	1.8% (*n* = 457)	4.1% (*n* = 982)	6.4% [42]
Filipino	1.5% (*n* = 366)	2.0% (*n* = 470)	2.6% [42]
First Nations, Inuit, Metis	1.2% (*n* = 298)	2.3% (*n* = 537)	3.9% [42]
Japanese	0.1% (*n* = 22)	0.1% (*n* = 23)	0.3% [42]
Korean	0.3% (*n* = 70)	0.6% (*n* = 141)	0.7% [42]
Latin American	1.7% (*n* = 432)	2.2% (*n* = 522)	2.4% [42]
Southeast Asian	0.7% (*n* = 182)	0.8% (*n* = 197)	1.8% [42]
West Asian	0.8% (*n* = 205)	1.0% (*n* = 243)	3.6% [42]
Arab	1.2% (*n* = 291)	1.4% (*n* = 333)	0.3% [42]
Did not report	32.7% (*n* = 8,212)	Less than 0.01% (*n* < 5)	-
**Healthcare worker**			
No	86.8% (*n* = 21,829)	83.8% (*n* = 19,937)	-
Yes	12.9% (*n* = 3,231)	13.1% (*n* = 3,111)	6.3% [43]
Did not report	0.3% (*n* = 85)	3.1% (*n* = 747)	-
**Post-secondary student**			
No	66.1% (*n* = 16,630)	52.0% (*n* = 12,367)	-
Yes	33.2% (*n* = 8,347)	13.2% (*n* = 3,137)	6.2% [42]
Did not report	0.7% (*n* = 168)	34.8% (*n* = 8,291)	-
**Referral type**			
Hospital/NLO	1.3% (*n* = 321)	0.4% (*n* = 105)	-
Self-referral	98.7% (*n* = 24,824)	99.6% (*n* = 23,690)	-
**Location of residence**			
Rural (less than 1 K)	2.9% (*n* = 738)	1.9% (*n* = 442)	13.8% [44]
Small population centres (1 K to 29,999)	17.4% (*n* = 4,364)	15.5% (*n* = 3,695)	10.0% [44]
Medium population centres (30 K to 99,999)	9.0% (*n* = 2,254)	10.7% (*n* = 2,546)	8.1% [44]
Large and urban population centres (100 K +)	65.4% (*n* = 16,44)	71.1% (*n* = 16,915)	68.2% [44]
Outside of Ontario	-	0.2% (*n* = 35)	-
Did not report	5.4% (*n* = 1,345)	0.7% (*n* = 162)	-
**Primary mental health issue as assessed by service provider**			
Alcohol	-	0.2% (*n* = 37)	-
Anxiety & depression	2.9% (*n* = 726)	-	-
Anxiety related to pandemic	6.3% (*n* = 1,583)	-	-
Chronic illness	-	0.1% (*n* = 31)	-
Chronic pain	-	0.7% (*n* = 168)	-
Depression	16.2% (*n* = 4,074)	30.9% (*n* = 7,360)	-
Generalized anxiety disorder	57.9% (*n* = 14,549)	38.5% (*n* = 9,159)	-
Grief and loss	1.1% (*n* = 267)	-	-
Health anxiety	-	0.8% (*n* = 199)	-
Insomnia	1.0% (*n* = 267)	1.2% (*n* = 281)	-
Obsessive compulsive disorder (OCD)	0.3% (*n* = 82)	-	-
Panic disorder	-	2.2% (*n* = 520)	-
Post-traumatic stress disorder	0.4% (*n* = 102)	10.2% (*n* = 2,419)	-
Social anxiety	1.4% (*n* = 339)	-	-
Social phobia	-	5.0% (*n* = 1,192)	-
Stress	-	10.2% (*n* = 2,429)	-
Trauma support	0.8% (*n* = 205)	-	-
No primary mental health issue identified	11.8% (*n* = 2,977)	-	-
**Baseline anxiety (GAD-7) (categorical)**			
Minimum (0–4)	5.2% (*n* = 1,294)	10.6% (*n* = 2,529)	-
Mild (5–9)	22.6% (*n* = 5,677)	27.7% (*n* = 6,596)	-
Moderate (10–14)	22.1% (*n* = 5,561)	27.5% (*n* = 6,531)	-
Severe (15–21)	36.6% (*n* = 9,240)	24.3% (*n* = 5,789)	-
No score provided	13.6% (*n* = 3,409)	9.9% (*n* = 2,350)	-
**Baseline depression (PHQ-9) (categorical)**			
Minimum (0–4)	11.1% (*n* = 2,790)	8.9% (*n* = 2,106)	-
Mild (5–9)	16.8% (*n* = 4,232)	24.1% (*n* = 5,730)	-
Moderate (10–14)	20.3% (*n* = 5,113)	27.2% (*n* = 6,470)	-
Moderately Severe (15–19)	21.4% (*n* = 5,373)	19.6% (*n* = 4,661)	-
Severe (20–27)	21.8% (*n* = 5,483)	10.4% (*n* = 2,468)	-
No score provided	8.6% (*n* = 2,154)	9.9% (*n* = 2,360)	-
**Duration in program (in weeks)**			
Mean (SD)	13 weeks (7.1)	10 weeks (4.1)	-
Range	0–65	0–57	-
**Messages sent by participant to therapist**			
Mean (SD)	5 messages (10.3)	13 messages (16.4)	-
Range	0-356	1-480	-
**Messages sent by therapist to participant**			
Mean (SD)	13 messages (17.1)	25 messages (20.7)	-
Range	0-174	1-794	-
**†Clinical hours (number of hours spent by therapist on each participant)**			
Mean (SD)	1.4 h (1.9)	-	-
Range	0–64	-	-

*Sexual and gender minorities is an umbrella category that included people who self-identified as transgender,* intersex*,* Two-spirit*,* or other undisclosed gender identity*

*†Clinical hours were only collected for LifeWorks AbilitiCBT*

### Baseline symptoms and measures of engagement

**Fig. 2:**
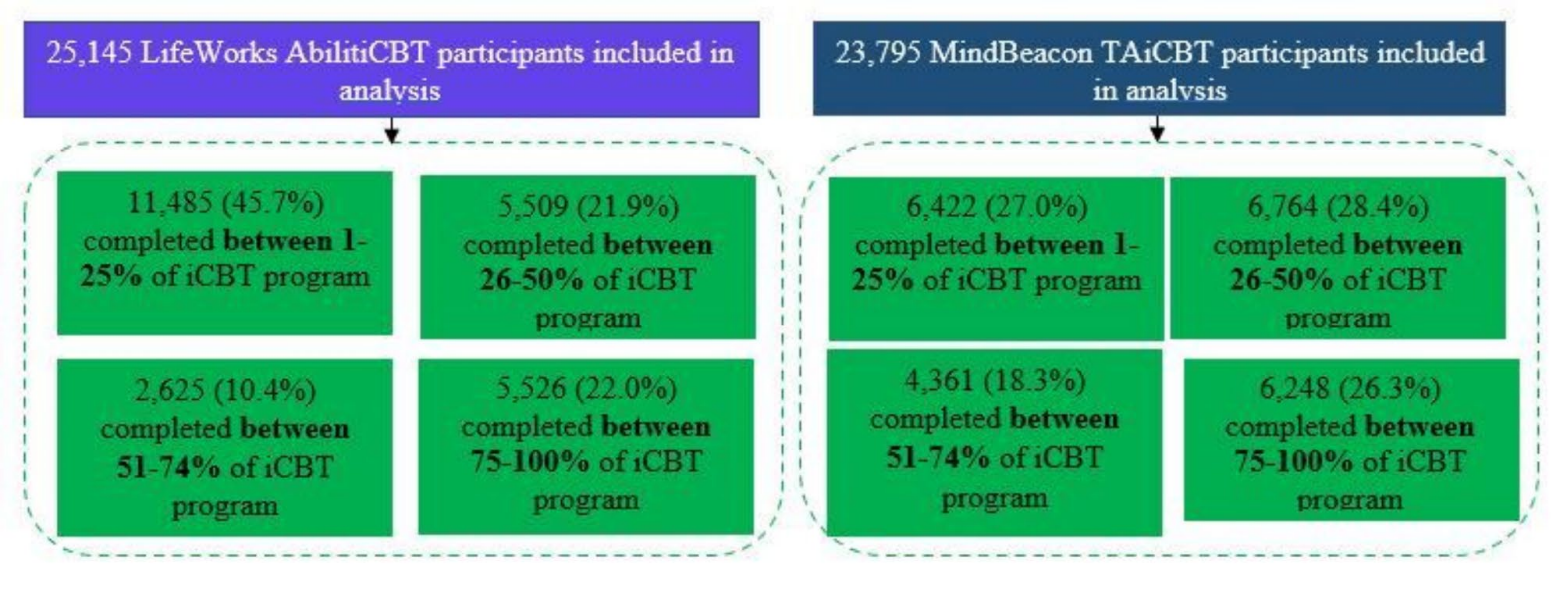
Program Completion. Showcases program completion rates for participants in LifeWorks AbilitiCBT and MindBeacon TAiCBT programs

As shown in Table 2, there was a large proportion of participants at baseline with severe anxiety symptoms, (36.6% of LifeWorks participants and 24.3% of MindBeacon participants). As well, 43.2% of LifeWorks and 30.0% of MindBeacon participants had moderately severe to severe depressive symptoms at baseline. Overall, there was greater baseline severity in anxiety and depression symptoms among LifeWorks participants (36.6%) compared to MindBeacon participants (24.3%). On average, LifeWorks participants spent 13 ± SD 7.1 weeks in the program and MindBeacon participants spent 10 ± SD 4.1 weeks in the program. There was greater message engagement between participants and therapists among MindBeacon participants compared to Lifeworks participants (sent by participant to therapist: 13 ± 17.1 SD versus 5 ± 10.3 SD messages; sent by therapist to participant: 25 ± 20.7 SD versus 13 ± 16.4 SD messages). In terms of direct therapist and participant engagement, LifeWorks therapists spent on average 1.4 ± 1.9 SD clinical hours on each participant during treatment. Clinical hours were not collected for MindBeacon participants. In Fig. 2. Program Completion, there was similar level of completion for all participants, 22.2% (*n* = 5,526) for LifeWorks participants and 26.3% (*n* = 6,248) for MindBeacon participants completing more than 75% of the iCBT program.

### Program outcomes

Table 3 reports estimated means and effect sizes between pre- and post- scores, percent changes, and reliable change in outcomes. Analysis revealed significant symptom reductions on all measures at post-treatment for both LifeWorks and MindBeacon. Hedges g effect sizes were calculated between pre-test to post-test scores. A small effect was observed on PHQ-9 scores (0.35) and GAD-7 scores (0.35) among LifeWorks AbilitiCBT participants and similarly for MindBeacon TAiCBT participants with PHQ-9 scores (0.34) and GAD-7 (0.32). The greatest percent change was in anxiety scores (17.4%) and depression scores (17.5%) for MindBeacon participants. A total of four GEE models were conducted to explore pre-post self-reported changes in depression and anxiety scores for LifeWorks and MindBeacon participants included in Table 4. GEE models revealed that change in depression scores were associated with greater program completion, higher baseline depression scores, more clinical hours spent by therapist for LifeWorks participants (*p* < 0.003) and more messages sent by therapists for MindBeacon participants (*p* < 0.000). GEE models revealed that change in anxiety scores were associated with greater program completion, higher baseline anxiety scores, more messages sent by LifeWorks participants (*p* < 0.002) and more messages sent by therapists for MindBeacon participants (*p* < 0.000). Regardless of program, greater program completion and more patient-therapist contact were associated with improvement in anxiety and depression scores.

**Table 3:** Pre-post change in GAD-7 and PHQ-9 scores for LifeWorks and MindBeacon participants that initiated iCBT between May 2020 and September 2021

	Estimated Marginal Means, Standard Deviations		Hedges g Effect Sizes from Pre-treatment	Percentage Change in Estimated Mean from Pre-treatment	Reliable Treatment Outcomes	
	Pre-treatment	Post-treatment	To Post-treatment	To Post-treatment	Deterioration	Recovery
**LifeWorks AbilitiCBT**						
Anxiety symptoms (GAD-7)	12.8 (5.5) *n* = 21,736	10.8 (5.7) *n* = 21,736	0.35 (0.34, 0.37)	15.5%	4.9% (*n* = 1,223)	25.5% (*n* = 4,298)
Depression symptoms (PHQ-9)	13.5 (7.2) *n* = 22,991	11.7 (7.1) *n* = 22,991	0.25 (0.23, 0.27)	13.3%	4.2% (*n* = 1,045)	21.6% (*n* = 3,444)
**MindBeacon TAiCBT**						
Anxiety symptoms (GAD-7)	10.9 (5.2) *n* = 21,445	9.1 (5.6) *n* = 21,445	0.32 (0.31, 0.34)	17.4%	6.6% (*n* = 1,578)	24.8% (*n* = 3,720)
Depression symptoms (PHQ-9)	12.0 (5.8) *n* = 21,435	9.9 (6.3) *n* = 21,435	0.34 (0.32, 0.36)	17.5%	3.2% (*n* = 761)	30.7% (*n* = 4,181)

*Based on the IAPT manual,* reliable deterioration was calculated based on all participants who have initiated iCBT. Reliable recovery was calculated from participants who initiated iCBT and who met clinical case criteria at baseline*

**Table 4:** Variables associated with pre-post change in GAD-7 and PHQ-9 scores (GEE models) for LifeWorks and MindBeacon participants that initiated iCBT between May 2020 and September 2021

		LifeWorks				MindBeacon			
		**Coefficient**	**Lower 95% CI**	**Upper 95% CI**	***p*** **-value**	**Coefficient**	**Lower 95% CI**	**Upper 95% CI**	***p*** **-value**
**GAD-7** ^*†*^	Intercept	1.174	1.093	1.255	0.000	0.632	0.538	0.726	0.000
	Pre GAD-7	0.834	0.829	0.839	0.000	0.848	0.843	0.854	0.000
	Program completion (categorical)								
	Less than 25% completion	Ref			0.000	Ref			
	Between 26–50% completion	-0.929	-0.999	-0.858	0.000	-0.413	-0.493	-0.332	0.000
	Between 51–74% completion	-1.633	-1.750	-1.560	0.000	-0.876	-0.965	-0.787	0.000
	More than 75% completion	-2.394	-2.518	-2.331	0.000	-1.327	-1.415	-1.238	0.000
	Time (categorical)				0.000				
	Pre	Ref			0.000	Ref			
	Post	1.989	1.938	2.05	0.000	1.829	1.767	1.890	0.000
	Messages sent by clients	0.005	0.001	0.008	0.002	0.0009	-0.002	0.004	0.487
	Clinical hours spent for LifeWorks **OR** Messages sent by therapists for MindBeacon*	-0.013	-0.031	0.005	0.160	-0.005	-0.007	-0.003	0.000
		**Coefficient**	**Lower 95% CI**	**Upper 95% CI**	**p-value**	**Coefficient**	**Lower 95% CI**	**Upper 95% CI**	***p*** **-value**
**PHQ-9** ^*§*^	Intercept	0.942	0.865	1.018	0.000	0.278	0.179	0.378	0.000
	Pre PHQ-9	0.864	0.860	0.868	0.000	0.873	0.868	0.878	0.000
	Program completion (categorical)								
	Less than 25% completion	Ref				Ref			
	Between 26–50% completion	-0.667	-0.746	-0.588	0.000	-0.468	-0.554	-0.382	0.000
	Between 51–74% completion	-1.462	-1.569	-1.355	0.000	-1.039	-1.134	-0.944	0.000
	More than 75% completion	-2.171	-2.275	-2.066	0.000	-1.531	-1.626	-1.436	0.000
	Time (categorical)								
	Pre	Ref				Ref			
	Post	1.767	1.701	1.834	0.000	2.130	2.066	2.196	0.000
	Messages sent by clients	0.003	-0.0007	0.006	0.119	0.001	-0.002	0.004	0.368
	Clinical hours spent for LifeWorks **OR** Messages sent by therapists for MindBeacon*	-0.032	-0.053	-0.018	0.003	-0.004	-0.006	-0.002	0.000

*For LifeWorks we included clinical hours spent in the GEE models and for MindBeacon we included messages sent by therapists

*†Upper quadrants are GEE models for change in GAD-7 scores*

*§Lower quadrants are GEE models for change in PHQ-9 scores*

## Discussion

In our uncontrolled observational study comparing LifeWorks AbilitiCBT and MindBeacon TAiCBT, we observed mild changes in anxiety and depression symptoms among all participants, which was associated with greater program completion, higher baseline anxiety and depression scores, and greater participant and therapist engagement, despite different therapist engagement offerings (i.e., synchronous & asynchronous vs. asynchronous). We also observed a similar demographic reach of participants in both programs among Ontario’s adult population, which were primarily women, middle-aged, predominantly white, self-referred, and living in a large urban area. At baseline, some participants among this sub-population were experiencing more severe levels anxiety or depression. In terms of engagement and program completion, participants from MindBeacon required more therapist engagement due to its asynchronous (messaging) model compared to participants in the LifeWorks program however, it should be noted that all participants had similar modest completion rates, with only approximately 20–25% of participants completing more than ¾ of their respective programs.

Noticeably, there was many individuals dropping out right after registering for the programs and during the intake assessment. Some plausible reasons for early dropout could be technical or platform difficulties, change in motivation, with participants potentially self-assessing their mental state at intake or determining the program was not suitable for themselves. Nevertheless, adherence to digital mental health therapies has been a longstanding issue, with adherence rates typically higher in study trials in comparison to real-world implementations and routine care [30]. A recent meta-analysis reviewing evidence from 64 randomised controlled trials on iCBT found adherence rates range from 6 to 100% [13]. In comparison, adherence rate for LifeWorks AbilitiCBT was 22.0% and MindBeacon TAiCBT was 26.3%. The variability in iCBT adherence and dropout is noticeable. Future work might consider determining participants that most likely benefit from iCBT from patient experience research and delve deeper into iCBT platform design and features that derive the most benefit in real-world implementation. Unlike randomized controlled trials, real-world implementation of iCBT does not closely control for and collect data on factors that could influence participant progress in the program e.g., motivators and facilitators to initiate iCBT, current psychotropic medication use, or prior and current use of other mental health services. In our study, for participants that disengaged from LifeWorks AbilitiCBT or MindBeacon TAiCBT, there was limited to no follow-up to divulge reasons for dropout and disengagement.

In terms of demographic reach for both LifeWorks and MindBeacon iCBT programs, the average age was 35.5 ± SD 12.3years old, which aligns with the increased prevalence of anxiety and depression among people in early to mid-adulthood in Canada during the pandemic [31, 32]. Only 22.4% of participants who initiated iCBT self-identified as men with previous research showing women having more positive perceptions of iCBT [33], while men had an increased risk of dropout and greater reluctance to seek help for mental health problems [33, 34]. Moreover, national surveys have reported that women had higher rates of screening positive for anxiety or depression compared to men during the pandemic [31, 32]. In line with our study, more women registered for iCBT through Lifeworks and MindBeacon programs, compared to men, while dropout rates were relatively similar among men and women in this study.

Although both programs were promoted to individuals experiencing mild to moderate depressive and/or anxiety symptoms, there was substantial severity at baseline among both programs with LifeWorks participants having greater severity in depression and anxiety symptoms at baseline. Interestingly, there were statistically significant changes observed in PHQ-9 and GAD-7 scores among program participants. For MindBeacon participants, more therapist messages significantly predicted change in PHQ-9 and GAD-7 scores. For Lifeworks participants, a greater number of clinical hours significantly predicted change in PHQ-9 scores and more participant messages significantly predicted change in GAD-7 scores. Some iCBT programs do not incorporate any form of contact with therapists, be it synchronous or asynchronous. Our findings demonstrate that increased contact with therapists correlates with greater clinical improvement over time implying that the presence of live therapists delivering messages is indeed a crucial component of iCBT programs.

The reliable recovery rates for LifeWorks AbilitiCBT (21.6–25.5%) and MindBeacon TAiCBT (24.8–30.7%) were lower compared to population-level digital mental health therapies in other jurisdictions like the Online Therapy Unit (36.3–45.0%), MindSpot (49.8–50.2%), NewAccess (64.0%) This Way Up (55.7%), but higher than IAPT (14.6–14.6%) when counting all participants who initiated treatment [14–18]. For fair comparisons, it is necessary to state programmatic differences between these real-world comparators. The Online Therapy unit offered a transdiagnostic course with five iCBT lessons [18], This Way Up and MindSpot offered a suite of low intensity therapies [14, 17], while IAPT and NewAccess are based on stepped care model with a mixture of low and high intensity therapies [15, 16]. MindBeacon participants exhibited statistically significantly (*p* < 0.001) higher rates of reliable recovery in PHQ-9 scores compared to LifeWorks participants, whereas the reliable recovery in GAD-7 scores between LifeWorks and MindBeacon participants did not statistically differ. This difference in PHQ-9 scores may be attributed to the nuanced programmatic approaches: LifeWorks AbilitiCBT uses standardized 10–12 modules, while MindBeacon TAiCBT offers personalized playlists tailored to mental health conditions and participants’ specific needs and preferences. The lower reliable recovery rates in both iCBT programs compared to some real-world comparators may stem from challenges like treatment adherence issues, heightened baseline severity, and potential gaps in integration into the larger mental health system. These iCBT implementations occurred in authentic, uncontrolled environments, introducing various confounding factors to reliable recovery rates.

### Implications for policy, research, and practice

While these iCBT programs were free and theoretically easy to access, there was a gradual dropout with a small subset engaging with the programs. Optimistically with many participants registering and completing an intake assessment for both programs may indicate peoples’ self-awareness to recognize and assess their mental health state during the pandemic. More importantly, participants with low program completion had pre-post self-reported change in outcomes which indicates participants’ personal preferences on the frequency and intensity with which they choose to engage with iCBT. Additionally with majority of participants entering through the self-referral mode of entry speaks to the low barrier access needed for digital mental health services to be broadly available to the population especially for individuals that are reluctant to access mental health services due to stigma.

Secondly, a considerable proportion of people with severe baseline anxiety and/or depression registered for the iCBT programs with varying levels of program completion. iCBT is a viable service option for patients with mild to moderate mental health disorders [10, 35], but individuals with higher symptom severity also engaged with these programs in the absence of other options [12]. The literature is mixed in determining whether higher baseline severity is a positive or negative predictor of symptom improvement, but people with severe baseline depression or anxiety symptoms had improvements in pre-post self-reported scores while participating in these two programs [36]. Program suitability for individuals with severe baseline symptoms should be decided on a case-by-case basis taking into consideration prior mental health history, psychotropic medication use, willingness to engage in an online program, and case complexity to determine if iCBT could be a standalone treatment option or offered in tandem with other treatment [37]. A broad entry criterion should be adopted to ensure that individuals that would benefit from iCBT including those with severe symptoms get access.

Thirdly, the cost-effectiveness of large publicly funded iCBT programs depends on the payment models and arrangements made between government and service providers. As seen in both programs, there was a high level of drop out during initial stages of registration and intake assessment, with participants dropping out steadily even after initiating iCBT. Both programs were front-loaded with a high reimbursement to LifeWorks and MindBeacon for completed intake assessments, which meant paying for assessments that did not lead to initiation and/or program completion or referral to other external programs if the eligibility and program suitability criteria for participants for iCBT were not met. Publicly funded models should consider how to economically distribute costs across registration, intake assessment and completing iCBT program components.

Lastly, both iCBT programs were standalone external services with little to no integration into the larger mental health system. Around 99.1% of participants were self-referred to the iCBT programs with less than 1% being referred from hospitals and mental health organizations in Ontario. The intake assessments for both programs identified when participants required higher levels of care, were of risk to themselves or others, had accessibility or technological limitations, or lacked engagement early in the program. But there was no formal referral pathway for people deemed not eligible or suitable for the programs to existing mental health services in the larger mental health system - instead external mental health services were suggested, and participants were required to follow up on their own with no data on if they did. This was further supported by a qualitative study exploring patient and therapist experiences with these programs [39]. It should be noted that the standalone nature of both programs allowed for easier implementation and scaling up in a short time span, especially in jurisdictions where publicly accessible mental health services are in its early stages. Optimally, having a more comprehensive intake process with integration to the mental health system could directly triage people to a range of appropriate mental health services depending on their unique and changing mental health needs.

### Limitations and strengths

As an uncontrolled observational study, there are several limitations and strengths. There was no active control group, so we cannot derive the true effectiveness of the programs. Future studies could use linkage to health administrative datasets to assess impact on overall health services utilization and compare outcomes to a control group that is matched on a variety of sociodemographic and clinical variables. However, it’s important to note some limitations in our approach. By not imputing missing values, our study may have introduced biases, including sampling bias and potentially decreasing statistical power. This approach could also lead to a biased estimate of parameters and a loss of valuable information on participants that were excluded from the analysis, thereby impacting the generalizability and precision of our results. Therefore, while our findings provide insights, they should be interpreted with caution considering these limitations. Additionally, participants in both iCBT programs may have simultaneously received medication, counselling, or other types of mental health care which may have contributed to improvement, but we do not have information on this from the data. Furthermore, a comprehensive account of the assessment process was not provided, and the programs did not confirm if mental health diagnosis was clinician-driven. It should also be noted that there may have been better suited psychiatric assessments beyond PHQ-9 and GAD-7 to measure change in symptoms for some of the primary mental health issues that were identified, which have impacted our psychological outcome findings. Lastly, paucity of data about reasons for dropout severely limits the interpretation and evaluation of the results. Direct comparisons between programs cannot be made without detailed data on the reasons for drop out which may or may not have been different between the two programs.

While there were limitations, this evaluation generated evidence on a population-level implementation of two iCBT programs with findings on the reach of both programs, levels of engagement and completion, and insights on improvement through change in PHQ-9 and GAD-7 scores. There are a number of strengths in this study including: (a) the total sample size of registered participants of both these programs is much larger than other iCBT programs like This Way Up, NewAccess Online Therapy Unit, (b) unlike other real world iCBT programs like This Way Up, MindSpot, NewAccess, IAPT, and Online Therapy Unit that were evaluated by respective program leads involved in program development and implementation, this evaluation was conducted by a neutral third-party evaluation team contracted by the provincial government, (c) apart from international comparators this is the largest iCBT implementation in Canada to date. Many of the findings and implications from this evaluation informed the successful integration of iCBT as a service offering into the Ontario Structured Psychotherapy (OSP) program which is coordinated by ten partner hospitals to offer iCBT across Ontario, Canada.

## Conclusion

Publicly funded iCBT was offered throughout the pandemic in Ontario, free of charge through two programs and expanded rapidly to deliver mental health support during a time of dire need. The scale up of these two iCBT programs was one of the largest population-level implementations of iCBT in Canada. Across both programs, participants were predominantly female, White, in their mid-30s, and residing in large to urban population centres. Only a small subset of participants completed 75% of iCBT treatment through either program. Yet our study emphasizes clinically significant pre-post self-reported changes: 26% of LifeWorks and 25% of MindBeacon participants demonstrated reliable recovery in GAD-7 scores, and 22% of LifeWorks and 31% of MindBeacon participants exhibited reliable recovery in PHQ-9 scores.

Moving ahead, MindBeacon TAiCBT with another iCBT program were integrated in the Ontario Structured Psychotherapy (OSP) program to provide iCBT to up to 9,000 individuals [38]. Evaluation results were reported to the provincial government on an ongoing basis and used to inform next steps. Ultimately, it is important to highlight what worked, for whom, and what could have been improved for future large-scale digital mental health interventions and evaluations. There were several publicly funded iCBT programs offered across Canada during the pandemic to offset mounting mental health concerns and increase access to care, but there are no publicly available data on their performance. Future iterations of iCBT should consider the following: (1) Acknowledging that low barrier mental health services with participant-led engagement and completion lead to reduction in symptoms, (2) Adopting a broad entry criteria as initial results suggest individuals with severe anxiety or depression reported improvement but needs to be substantiated with more research, (3) Considering the high level of drop out after intake assessment, different funding models should be adopted to separate comprehensive assessment from single program intake so people can be directed to more suitable resources and incentivize retention and (4) Integrating and building seamless connectivity of iCBT programs to the larger mental health care system.

## Data Availability

The data that support the findings of this study are available on request from the corresponding author, B.K. The data are not publicly available due to restrictions whereby including that could compromise the privacy of research participants.
